# Supplementary material for: Causal Relationship between Aging and Anorexia Nervosa: A White-Matter-Microstructure-Mediated Mendelian Randomization Analysis
Source: Biomedicines. 2024 Aug 16;12(8):1874. doi: 10.3390/biomedicines12081874 (PMC11351342; doi:10.3390/biomedicines12081874)
Supplement: Supplementary file 1 [file biomedicines-12-01874-s001.zip › Supplementary Figure/Figure S4/Singe SNP plot of MR analysis for TL to ubm-b-1714.pdf]

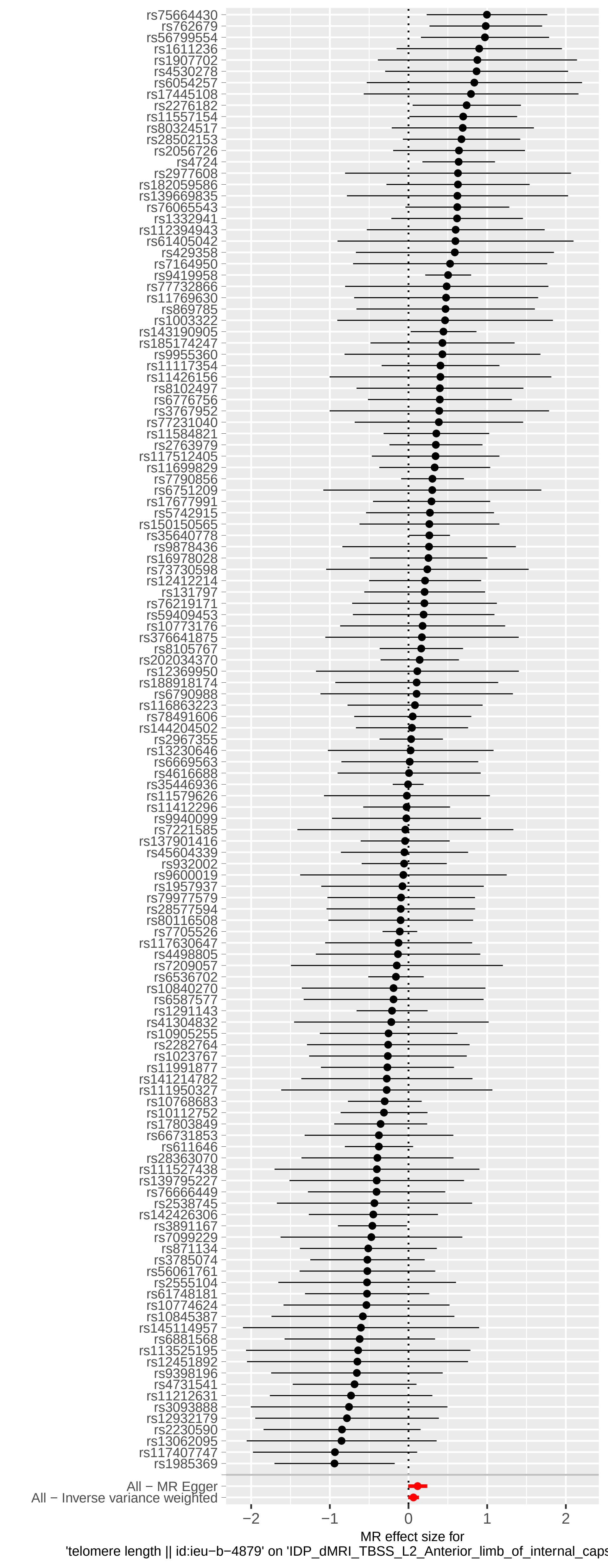
rs75664430
rs762679
rs56799554
rs1611236
rs1907702
rs4530278
rs6054257
rs17445108
rs2276182
rs11557154
rs80324517
rs28502153
rs2056726
rs4724
rs2977608
rs182059586
rs139669835
rs76065543
rs1332941
rs112394943
rs61405042
rs429358
rs7164950
rs9419958
rs77732866
rs11769630
rs869785
rs1003322
rs143190905
rs185174247
rs9955360
rs11117354
rs11426156
rs8102497
rs6776756
rs3767952
rs77231040
rs11584821
rs2763979
rs117512405
rs11699829
rs7790856
rs6751209
rs17677991
rs5742915
rs150150565
rs35640778
rs9878436
rs16978028
rs73730598
rs12412214
rs131797
rs76219171
rs59409453
rs10773176
rs376641875
rs8105767
rs202034370
rs12369950
rs188918174
rs6790988
rs116863223
rs78491606
rs144204502
rs2967355
rs13230646
rs6669563
rs4616688
rs35446936
rs11579626
rs11412296
rs9940099
rs7221585
rs137901416
rs45604339
rs932002
rs9600019
rs1957937
rs79977579
rs28577594
rs80116508
rs7705526
rs117630647
rs4498805
rs7209057
rs6536702
rs10840270
rs6587577
rs1291143
rs41304832
rs10905255
rs2282764
rs1023767
rs11991877
rs141214782
rs111950327
rs10768683
rs10112752
rs17803849
rs66731853
rs611646
rs28363070
rs111527438
rs139795227
rs76666449
rs2538745
rs142426306
rs3891167
rs7099229
rs871134
rs3785074
rs56061761
rs2555104
rs61748181
rs10774624
rs10845387
rs145114957
rs6881568
rs113525195
rs12451892
rs9398196
rs4731541
rs11212631
rs3093888
rs12932179
rs2230590
rs13062095
rs117407747
rs1985369
All − MR Egger
All − Inverse variance weighted
−2
−1
0
1
2
MR effect size for
'telomere length || id:ieu−b−4879' on 'IDP_dMRI_TBSS_L2_Anterior_limb_of_internal_caps
